# A Smartphone App to Promote Patient Activation and Support Shared Decision-making in People With a Diagnosis of Schizophrenia in Outpatient Treatment Settings (Momentum Trial): Randomized Controlled Assessor-Blinded Trial

**DOI:** 10.2196/40292

**Published:** 2022-10-26

**Authors:** Tobias Vitger, Carsten Hjorthøj, Stephen F Austin, Lone Petersen, Esben Sandvik Tønder, Merete Nordentoft, Lisa Korsbek

**Affiliations:** 1 Competence Center for Rehabilitation and Recovery Mental Health Center Ballerup Mental Health Services in the Capital Region of Denmark Ballerup Denmark; 2 Department of Clinical Medicine University of Copenhagen Copenhagen Denmark; 3 Copenhagen Research Center for Mental Health – CORE Mental Health Center Copenhagen Copenhagen University Hospital Copenhagen Denmark; 4 Section of Epidemiology Department of Public Health University of Copenhagen Copenhagen Denmark; 5 Psychiatric Research Unit Psychiatry Region Zealand Slagelse Denmark; 6 The Mental Health Services of Zealand Slagelse Denmark; 7 The Mental Health Centre Odense Mental Health Services in the Region of Southern Denmark Odense Denmark

**Keywords:** mobile health, mHealth, digital intervention, shared decision-making, patient activation, schizophrenia, schizotypal, early intervention, randomized clinical trial, mobile phone

## Abstract

**Background:**

Shared decision-making (SDM) is a process aimed at facilitating patient-centered care by ensuring that the patient and provider are actively involved in treatment decisions. In mental health care, SDM has been advocated as a means for the patient to gain or regain control and responsibility over their life and recovery process. To support the process of patient-centered care and SDM, digital tools may have advantages in terms of accessibility, structure, and reminders.

**Objective:**

In this randomized controlled trial, we aimed to investigate the effect of a digital tool to support patient activation and SDM.

**Methods:**

The trial was designed as a randomized, assessor-blinded, 2-armed, parallel-group multicenter trial investigating the use of a digital SDM intervention for 6 months compared with treatment as usual. Participants with a diagnosis of schizophrenia, schizotypal or delusional disorder were recruited from 9 outpatient treatment sites in the Capital Region of Denmark. The primary outcome was the self-reported level of activation at the postintervention time point. The secondary outcomes included self-efficacy, hope, working alliance, satisfaction, preparedness for treatment consultation, symptom severity, and level of functioning. Explorative outcomes on the effect of the intervention at the midintervention time point along with objective data on the use of the digital tool were collected.

**Results:**

In total, 194 participants were included. The intention-to-treat analysis revealed a statistically significant effect favoring the intervention group on patient activation (mean difference 4.39, 95% CI 0.99-7.79; Cohen *d*=0.33; *P*=.01), confidence in communicating with one’s provider (mean difference 1.85, 95% CI 0.01-3.69; Cohen *d*=0.24; *P*=.05), and feeling prepared for decision-making (mean difference 5.12, 95% CI 0.16-10.08; Cohen *d*=0.27; *P*=.04). We found no effect of the digital SDM tool on treatment satisfaction, hope, self-efficacy, working alliance, severity of symptoms, level of functioning, use of antipsychotic medicine, and number or length of psychiatric hospital admissions.

**Conclusions:**

This trial showed a significant effect of a digital SDM tool on the subjective level of patient activation, confidence in communicating with one’s provider, and feeling prepared for decision-making at the postintervention time point. The effect size was smaller than the 0.42 effect size that we had anticipated and sampled for. The trial contributes to the evidence on how digital tools may support patient-centered care and SDM in mental health care.

**Trial Registration:**

ClinicalTrials.gov NCT03554655; https://clinicaltrials.gov/ct2/show/NCT03554655

**International Registered Report Identifier (IRRID):**

RR2-doi: 10.1186/s12888-019-2143-2

## Introduction

### Shared Decision-making

Shared decision-making (SDM) is a collaborative process between ≥2 partners. In a health care setting, SDM is often designated to be between a patient and provider. It is a continuous cycle aimed at facilitating patient-centered care and making joint treatment decisions. In mental health care, SDM has been proposed as a means to contribute to recovery-oriented care by inviting the patient to have more control and be more involved in their treatment decisions [1].

The current evidence on the effectiveness of SDM in mental health care is somewhat inconclusive but appears to be promising. Studies have found that SDM interventions improve self-perceived involvement in decision-making [2], satisfaction [2], therapeutic alliance [2], decision self-efficacy [3], and adherence to pharmacological treatment [4].

Incorporating SDM into daily practice in mental health care has shown to face some of the same barriers as recovery-oriented interventions, such as changing health care professionals’ paternalistic approaches, beliefs that SDM is time consuming and inappropriate for patients with severe mental illness, or discrepancies between the patients’ needs and values versus the goals and values of the health care provider and the organization [5,6].

To address these barriers, providers are encouraged to consider the patients’ decision-making skills, talk to the patient about how they prefer a decision process to be, and incorporate tools to support the SDM process [7]. In addition, activating patients may also support SDM; active patients who seek collaborative care could also activate their provider, resulting in a good foundation for SDM [8]. Much research has been conducted on patient activation with the conceptualization that active patients consider their own role in the treatment to be important, are engaged in managing their own health and care, feel confident when collaborating with their provider, and have the knowledge and skills to manage their condition [9]. The ability to maintain these behaviors even during stressful times is believed to characterize a patient with high levels of activation.

### Digital Tools to Support SDM

To support SDM while using the continuous development and use of digital solutions, researchers have started to investigate how digital interventions may support SDM. Digital mental health interventions, such as interventions including a smartphone app, have been found to significantly outperform control groups [10]. However, the evidence on digital mental health interventions to support SDM is sparse, but a recent meta-analysis found that digital SDM interventions may have an effect on patient activation, decisional conflict, working alliance, and severity of general symptoms [11]. The meta-analysis also concluded that while digital interventions to support SDM are promising, the limited evidence is in need for quality research.

This study aimed to provide new evidence on the effectiveness of a digital SDM intervention in mental health care and strengthen the evidence on how digital tools may promote patient activation. We evaluated the effectiveness of a digital solution to support SDM in an outpatient setting for people diagnosed with schizophrenia. We hypothesized that the intervention would support SDM, resulting in higher levels of self-perceived patient activation. With higher levels of patient activation, we also expected to see improvements in working alliance, hope, self-efficacy, satisfaction, feeling prepared for decision-making, confidence in communicating with one’s provider, severity of symptoms, level of functioning, number of hospitalizations, and adherence.

## Methods

### Trial Design and Setting

This study was a 2-arm, assessor-blinded, randomized parallel-group trial conducted in 9 outpatient treatment sites called OPUS in the Capital Region of Denmark. OPUS is a 2-year treatment program providing specialized early intervention treatment to patients with a debuting diagnosis of schizophrenia or related psychotic disorders in the age group of 18 to 35 years in Denmark. This trial compared a control group receiving treatment as usual (TAU) with an intervention group receiving a smartphone app as a supplement to TAU. The participants were recruited between January 2019 and March 2021. Assessments were conducted at baseline, 3 months after baseline (midintervention time point), and 6 months after baseline (postintervention time point). Detailed information on the trial design and methodology of the study is available in the study protocol [12].

### Participants and Eligibility Criteria

Eligible patients were referred to the study by their primary providers. Patients were eligible for inclusion if they were receiving treatment in OPUS (see the section *Treatment as Usual* for information on OPUS), had at least 6 months left of their OPUS program, access to a smartphone, and understood Danish. Patients were enrolled after meeting a staff member from the research team who provided detailed verbal and written information about the study, and written consent was obtained.

### Randomization and Blinding

Participants were randomized with an even allocation of 1:1 to either the intervention group (TAU plus app) or the control group (TAU minus app). Randomization was performed after completion of the baseline assessment. Block randomization was used to achieve balance in the allocation of participants to both treatment arms. The block sizes were randomly altered among 2, 4, and 6. The block sizes were concealed from the researchers during recruitment. The nonstratified randomization sequence was computerized and facilitated by the Odense Patient Data Explorative Network (OPEN) to ensure allocation concealment. The concealment was kept digital at OPEN until data collection ended and data analysis began. To ensure blinding of the data analyst, OPEN provided information on which group participants had been part of but without labeling the groups. This way the data analysis could be performed without bias by knowing who had been in the control group and who had been in the intervention group. After the whole research group had accepted the results of the data analysis and conclusions had been drawn, OPEN was contacted to reveal the labeling of the 2 groups.

Researchers collecting and analyzing data were blinded, but given the nature of the intervention, patients and health care providers were not blinded. All patients were at each visit, with the researcher thoroughly instructed not to mention anything about their randomization allocation. Therefore, all questionnaire outcomes (answered by the patient or provider) were not blinded, whereas the interview outcomes (assessor-rated) were blinded.

### Interventions

#### Treatment as Usual

Participants randomized to the control group continued with TAU and did not receive the digital SDM intervention. TAU in this trial was provided by OPUS, a treatment facility offering specialized early intervention by combining three key elements: (1) assertive community treatment aimed at maintaining or developing the patient’s coping skills and integration in society; (2) family involvement through multifamily groups and single-family sessions; and (3) social skills training to support patients with impaired social skills [13]. Patients starting OPUS are assigned to a primary provider with weekly sessions (excluding group sessions) lasting for approximately 40 to 60 minutes. Primary providers in OPUS may have a background as a psychologist, nurse, social worker, physiotherapist or vocational therapist. The OPUS treatment does facilitate recovery and SDM elements with its patient-centered approach where patients are considered *a long-awaited guest who should feel at home during a visit* and who are encouraged to take an active part in the treatment. Nevertheless, we chose to conduct the study in OPUS because the results from our pilot study indicated that younger adults with schizophrenia spectrum disorders showed a positive attitude toward using a digital tool to support their care [14]. During recruitment for the trial, the providers had approximately 15 patients at a time and were able to have patients in both groups.

The study protocol provides more information on TAU [12].

#### The Intervention Group

Before the trial, we developed a digital SDM tool for the process of cocreation among patients, providers, and researchers, with preparation for treatment consultation as the main function. A pilot study revealed that the tool was perceived to be useful with relevant content by patients and providers [14]. On the basis of feedback from the pilot study, the app was adjusted accordingly and included a new functionality, an option to perform a daily self-assessment.

**Figure 1:**
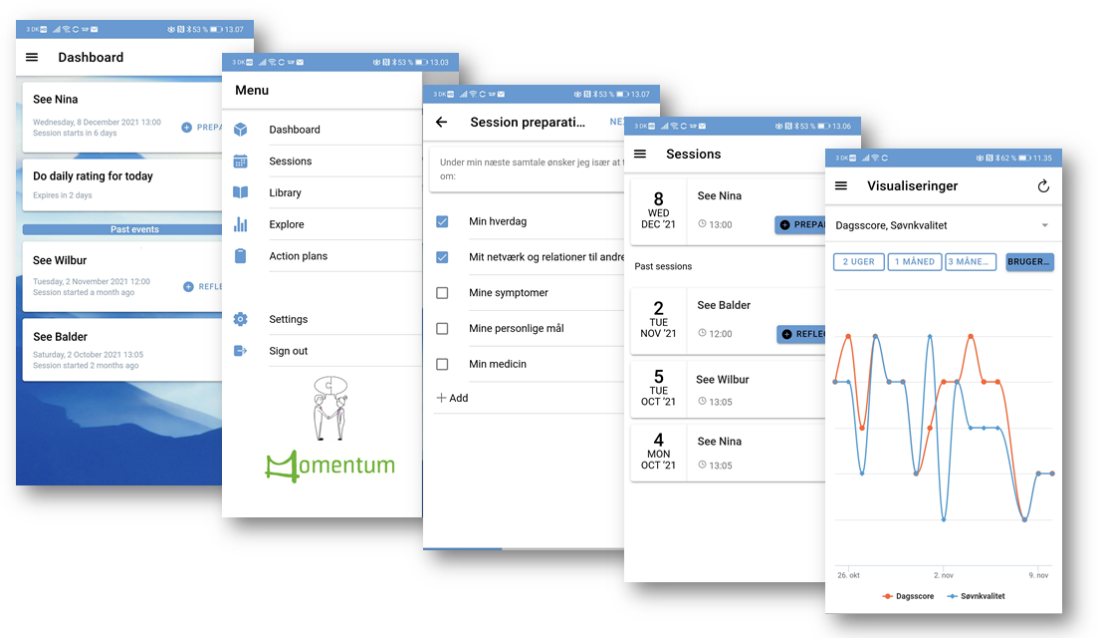
Digital shared decision-making tool for smartphones.

The intervention group continued with TAU and was invited to use the digital system provided by the IT company Monsenso. The digital SDM tool tested in this trial consisted of a smartphone app for the patient with functions, such as preparation for consultation, daily self-assessments, action plans, and educational material. The app was synchronized to a web portal that the patient’s provider could access before the consultation. The intention was that the patient could use the app outside of the consultation and that the provider before an upcoming consultation could become aware of what the patient would like to address at the consultation while also seeing how the patient had scored themselves on the self-assessments. The patients were encouraged to use the app daily or what felt meaningful. Patients were shown how to set up reminders within the app to enable push messages. Enabling these push messages was voluntary. Providers were encouraged to use the web portal before a consultation; however, there were no options for reminders or push messages for the providers. Most importantly, patients and providers were encouraged to discuss how best to use the system and how to incorporate it to support the consultations. The digital system is illustrated in Figure 1.

### Outcomes

#### Baseline Parameters

Information on the characteristics of both patients and providers was collected. As preferences in clinical decision-making have been found to be related to patient involvement, the Clinical Decision Making Style (CDMS) questionnaire was completed by both the patient and provider at baseline. The questionnaire consisted of 2 subscales: preference for participation in decision-making and preference for receiving information. This questionnaire was only completed at baseline because research indicates that CDMS scores are stable over 12 months [15].

#### Primary Outcome

Our primary outcome was the difference in self-perceived patient activation between the groups at the postintervention time point, as measured by the 10-item Consumer Health Activation Index for mental health (CHAI-MH) [16].

#### Secondary Outcomes

Our secondary outcomes consisted of questionnaires completed by participating patients and providers, a clinical interview, and data from the Danish National Patient Register. Patients completed the following questionnaires: self-perceived feeling of hope and optimism measured by the 6-item Adult State Hope Scale [17], self-efficacy measured by the 10-item General Self-Efficacy Scale (GSE) [18], confidence in communicating with one’s provider measured by the 5-item Perceived Efficacy in Patient-Physician Interactions (PEPPI) Questionnaire [19], therapeutic alliance between the patient and provider measured by the 12-item Working Alliance Inventory–short form (WAI-S) [20], feeling prepared to make a treatment decision by the 10-item Preparation for Decision-Making (PrepDM) [21], and satisfaction with treatment measured by the 8-item Client Satisfaction Questionnaire (CSQ) [22]. In addition, a clinical interview was conducted to assess the participants’ positive and negative symptoms, together with their level of functioning. We used the Scale for the Assessment of Positive Symptoms (SAPS) [23], Scale for the Assessment of Negative Symptoms (SANS) [23], Global Assessment of Functioning (GAF) [24] and Personal and Social Performance Scale (PSP) [25]. A blinded researcher conducted the interviews. Providers completed 2 questionnaires for each of their patients participating in the trial: the therapeutic alliance between the provider and patient measured by the 12-item WAI–S [20] and the patient’s engagement measured by the Service Engagement Scale (SES)—collaboration subscale [26]. Finally, we collected data for all participating patients from the Danish National Patient Register-Psychiatry on the following: number of hospital admissions, length of admissions in days, and adherence to OPUS appointments. Reasoning for choosing the outcomes can be found in the study protocol.

#### Explorative Outcomes

To explore the acceptance and perceived usefulness of the smartphone app, participants in the intervention group completed the 4-item App Rating Questionnaire and the 4-item Mobile App Rating Scale—subscale subjective quality rating at the postintervention time point [27,28]. In addition, objective data on the use of the system (user sessions per day, screen views per day, screens per session, session duration and session instances, and user retention) were provided by Monsenso.

### Sample Size

As stated in our protocol, a sample size of 180 participants was estimated to be needed to detect a significant difference between the intervention and control groups, with an effect size of 0.42 on the CHAI-MH scale. The effect size was calculated based on previous research that measured patient activation, as described in the research protocol. For both the primary and secondary outcomes, a power of 80% and an α of .05 was chosen to reject the null hypothesis that the population means of the 2 groups are equal. Before recruitment, we estimated that 30% would be lost to follow-up (ie, not responding to contact at the postintervention time point). To adjust for this, a sample size of 260 participants is needed. However, during the recruitment of the first 100 participants, only 7 (7%) were lost to follow-up. As the rate was significantly lower than anticipated, we changed our estimated percentage of lost-to-follow-up from 30% to 7%, resulting in a required sample size of 194 participants.

### Statistical Methods

For the statistical analysis, the principles of intention to treat (ITT) were followed with a 2-tailed level of significance for all statistical tests set at .05. Analyses were performed using SAS Enterprise Guide 7.1. Differences in patient characteristics between the 2 groups were assessed using the 2-sample *t* test (2-tailed), chi-square test, and Fisher exact test (for variables with <5 observations). Generalized linear mixed effects regression analyses were performed to assess the 6-month intervention. A binary logistic regression was performed to evaluate the impact of the intervention on participants’ use of antipsychotic medication. Negative binomial regression was performed for count outcomes to estimate the incidence rate ratios on the number of hospitalizations and the length of hospitalization at the postintervention time point based on the group allocation. To handle missing data, we created and analyzed 100 imputed data sets using multiple imputations by chained equations using the group variable (*intervention* and *control*); the use of antipsychotic medicine variable; completed interview at the postintervention time point; and the participants’ baseline, midintervention, and postintervention scores. The use of antipsychotic medication at baseline (score=yes or no) was used as a variable for the imputed data sets due to a significant difference between groups in the use of antipsychotic medication at baseline. During data analyses, we found that participants who completed the postintervention interview scored lower on the CHAI-MH than participants who had not completed the interview. Although there were no between-group differences, we decided to include this dichotomous variable when computing multiple imputations for the questionnaire outcomes. For the imputed data sets on the interview outcomes, we did not use this variable because its value would be the same for all imputed data. Outcome scales with partially missing values were regarded as completely missing. For each outcome, an estimate of the effect was calculated for each imputed data set and finally combined using the Rubin rule. We also performed a complete case analysis for comparative purposes. The midintervention assessment was included for explorative purposes to assess whether a potential effect occurred before or after 3 months of intervention.

### Ethical Considerations

The trial was approved by the Regional Ethics Committee in the Capital Region of Denmark under file number H-17025550 and the Knowledge Centre on Data Protection Compliance (Videnscenter for Dataanmeldelser) under approval number P-2019-502. The trial was registered at ClinicalTrials.gov under the identifier NCT03554655. No economic compensation was provided for participation.

### Changes From the Protocol

As stated in our study protocol, we were interested in evaluating the mean duration per session for which the participants used the smartphone app. However, due to technical limitations, we were unable to assess the duration for which the participants used the app.

Owing to the COVID-19 pandemic, several assessments were conducted on the web; however, no statistically significant differences in scores for participants being assessed physically or virtually were detected.

Owing to a fire accident at OVHcloud (a global cloud service provider that stores Monsenso’s data), the digital system became unavailable for approximately 1 month during which participants were unable to access the app and web portal. This downtime affected approximately 36 participants in the intervention group. Owing to blinding, the research group did not directly reach out to participants. Instead, all providers were contacted regarding this issue and instructed to inform participants of the system being unavailable in the intervention group. After the system became available again, providers were instructed to inform participants to use the app again. In addition to the accident, a failure in the Monsenso back-up system resulted in a loss of data for the last month leading up to the fire accident. To assess whether the interruption had an impact on the use of the system, objective data on the use of the system before the accident were compared with data on the use after the system became available again.

In our study protocol, we calculated Cohen κ for the CDMS questionnaire to assess the level of agreement between patients and providers. However, due to the data structure of the CDMS, this was not possible, and we instead performed a *t* test to assess if there was a statistically significant difference between the responses of patients and providers.

Although we planned to assess the effect of the intervention based on duration in OPUS (eg, patients at the beginning of their treatment versus those at the end of their treatment), we were unable to do so because of safety procedures regarding merging patient-reported outcome data with data from the Danish National Health Registers.

## Results

### Overview

**Figure 2:**
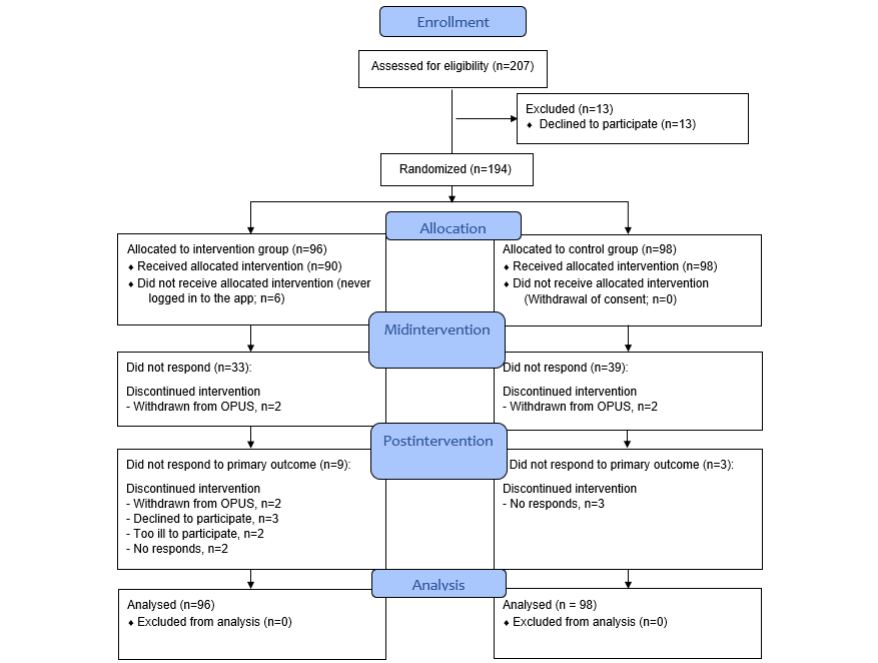
Flow diagram for the Momentum Trial.

Figure 2 presents the CONSORT (Consolidated Standards of Reporting Trials) flow diagram for the participants in the Momentum Trial. In total, 194 participants were included and randomized, with 98 to the control group and 96 to the intervention group. Recruitment began in January 2019, and the last patient was enrolled in September 2021.

### Background Characteristics

Tables 1 and 2 present the sociodemographics and background characteristics of patients and providers, respectively. The intervention and control groups differed in terms of age and use of antipsychotic medication, while no between-group differences were observed in gender, diagnosis, relationship status, level of education, employment status, duration of received treatment in OPUS at baseline, or scores on the CDMS questionnaire.

We also assessed the level of agreement between patients and providers on the WAI-S and CDMS. We observed a significant difference between the patients and providers on the information subscale (mean difference 0.33, 95% CI 0.18-0.46; *P*<.001) and on the decision-making subscale (mean difference −0.20, 95% CI −0.27 to −0.12; *P*<.001). While the mean differences are rather small, the results suggest that patients have a higher desire to be provided with information than providers’ desire to provide information. In contrast, patients have a smaller desire to be active participants in decision-making compared with the providers’ desire for active participation from the patient.

For the WAI-S scale, we considered an agreement between the patient and provider if their scores were within 12 points. With this range of agreement, the weighted Cohen κ was calculated to be 0.45 at baseline and 0.43 at the postintervention time point, indicating a stable yet weak level of agreement between patients and their provider on the working alliance.

There were no between-group differences on the WAI-S or the CDMS questionnaires.

**Table 1:** Sociodemographics and background characteristics of patients.

			Control group (n=98)		Intervention group (n=96)		Overall (N=194)
Age (years), mean (SD)			24.3 (4.3)		22.7 (3.7)		23.5 (4.1)
**Gender, n (%)**							
	Woman	55 (56.1)		65 (67.7)		120 (61.9)	
	Man	39 (39.8)		26 (27.1)		65 (33.5)	
	Nonbinary	4 (4.1)		5 (5.2)		9 (4.6)	
**Diagnosis, n (%)**							
	Schizophrenia	40 (40.8)		28 (29.2)		68 (35.1)	
	Schizotypal	48 (49.0)		54 (56.3)		102 (52.6)	
	Other nonorganic psychosis	8 (8.2)		14 (14.6)		22 (11.3)	
	Schizoaffective	2 (2.0)		0 (0)		2 (1.0)	
**In a relationship, n (%)**							
	Not in a relationship	54 (55.1)		46 (47.9)		100 (51.5)	
	In a relationship	44 (44.9)		50 (52.1)		94 (48.5)	
**Level of education, n (%)**							
	Primary school not completed	2 (2.0)		1 (1.0)		3 (1.5)	
	Primary school completed	29 (29.6)		34 (35.4)		63 (32.5)	
	High school or higher completed	67 (68.4)		61 (63.5)		128 (66)	
**Employment status, n (%)**							
	Employed	15 (15.3)		11 (11.5)		26 (13.4)	
	Student	34 (34.7)		47 (49.0)		81 (41.8)	
	Unemployed and not a student	49 (50.0)		38 (39.6)		87 (44.8)	
**Use of antipsychotics, n (%)**							
	Yes	76 (77.6)		57 (59.4)		133 (68.6)	
	No	22 (22.4)		39 (40.6)		61 (31.4)	
**Clinical Decision Making Style questionnaire score, mean (SD)**							
	Information	3.3 (0.5)		3.3 (0.6)		3.3 (0.5)	
	Participation in decision-making	2.1 (0.3)		2.1 (0.3)		2.1 (0.3)	
Duration of received treatment in OPUS at baseline (days), mean (SD)			306.52 (150.41)		262.10 (149.84)		284.54 (151.38)

**Table 2:** Sociodemographics and background characteristics of providers.

		Overall (n=76)
Age (years), mean (SD)		43.1 (10.1)
**Gender, n (%)**		
	Woman	66 (87.0)
	Man	10 (13.0)
Experience (years), mean (SD)		13 (8.2)
**Education of provider, n (%)**		
	Nurse	30 (39.5)
	Occupational therapist	15 (19.7)
	Psychologist	13 (17.1)
	Social worker	11 (14.5)
	Pedagogue	2 (2.6)
	Other	4 (5.3)
	Missing	1 (1.3)
**Clinical Decision Making Style questionnaire score, mean (SD)**		
	Information	3.0 (0.5)
	Participation in decision-making	2.3 (0.3)

### Use of Intervention

On the basis of objective data from Monsenso, only 86 participants used the app, meaning that 10 never started using the app despite being invited to use it. Although the reasons for this were not explored, there were reports of technical limitations where the participants’ phones did not support the app.

Owing to the fire accident at OVHcloud, we encountered a month in which the app was not accessible. To evaluate the impact of a “pause” in the intervention, we compared app use for the last 3 months leading up to the fire accident with data for the 3 months after the system became available again. We observed that 47% (17/36) of active users did not log back into the app, and there was a decrease in the number of app sessions from 1260 to 491 (61%), indicating that the fire accident had an impact on the participants’ use of the app.

### Lost-to-Follow-up

A total of 8.2% (16/194) of participants did not participate at the postintervention time point (11 participants from the intervention group and 5 from the control group). The difference in lost-to-follow-up between groups was mainly due to 4 participants in the intervention group who ended their OPUS treatment prematurely and 3 participants who refused to participate at the postintervention time point (vs 2 and 0 participants in the control group, respectively). The most frequent reason for loss to follow-up in the control group was not responding to contact (3 participants). According to our power calculation, we required 180 participants who completed the baseline and postintervention assessments to reach an adequate level of power of 0.42. We enrolled 178 participants who completed both baseline and postintervention assessments; therefore, we did not reach the level of power we had aimed at.

There was a large discrepancy between the completed questionnaires and the completed interviews at the postintervention time point. In the intervention group, 89% (85/96) completed at least 1 questionnaire, while 56% (54/96) completed the interview. In the control group, 95% (93/98) pf participants completed at least 1 questionnaire, while 70% (69/98) completed the interview.

The percentage of missing values across the 11 outcome measurements for each participant varied from 0% to 3% at baseline, and from 8% to 36% at the end of the intervention. In total, 52.4% of the records were incomplete, meaning that they had ≥1 missing variables at baseline or after the intervention. The variables with the highest proportion of missing information when events were combined were clinical interview data (SAPS, SANS, GAF, and PSP), where approximately 46% were missing. For the questionnaire variables, the highest proportion of missing data was found for the CSQ and PrepDM (approximately 40% missing data).

### Intention to Treat

**Figure 3:**
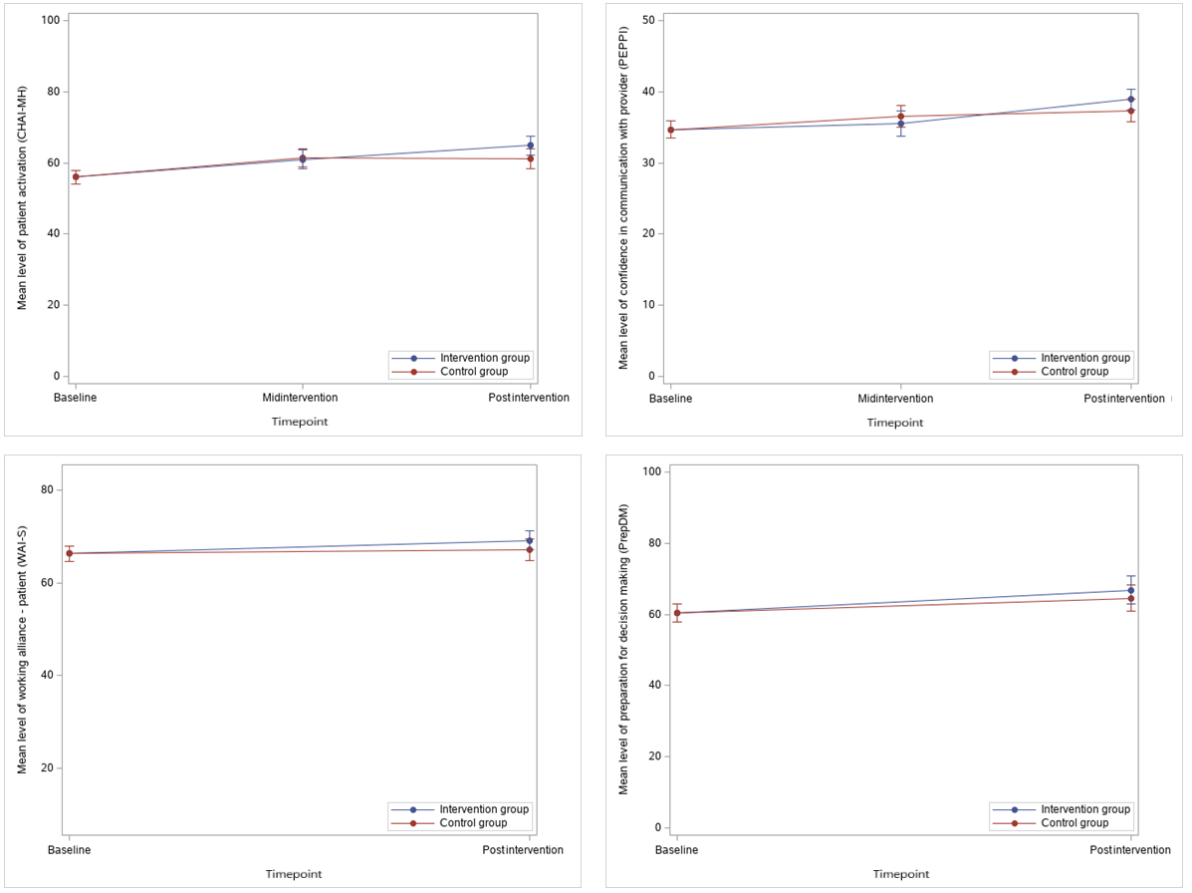
Mean scores on the primary outcome (Consumer Health Activation Index for mental health [CHAI-MH]) and selected secondary outcomes; Perceived Efficacy in Patient-Physician Interactions (PEPPI), PrepDM, and Working Alliance Inventory–short (WAI-S) for intervention and control groups over time with 95% CIs.

Tables 3 and 4 show the results of the mid- and postintervention ITT analyses, while Figure 3 illustrates the effect of the intervention. The Momentum Trial resulted in a statistically significant difference between the intervention and control groups in our primary outcome, CHAI-MH (mean difference 4.39, 95% CI 0.99-7.79; Cohen *d*=0.33; *P*=.01), favoring the intervention group. For the secondary outcomes, there were 2 scales with a minor statistically significant difference: PEPPI (mean difference 1.85, 95% CI 0.01-3.69; Cohen *d*=0.24; *P*=.05) and PrepDM (mean difference 5.12, 95% CI 0.16-10.08; Cohen *d*=0.27; *P*=.04), both favoring the intervention group. For the remaining outcome we found no statistically significant differences between the groups: Hope (mean difference 1.66, 95% CI −0.44 to 3.75; Cohen *d*=0.20; *P*=.12), GSE (mean difference 1.12, 95% CI −0.32 to 2.57; Cohen *d*=0.19; *P*=.13), WAI-S (mean difference 2.43, 95% CI −0.25 to 5.12; Cohen *d*=0.22; *P*=.08), CSQ (mean difference 0.89, 95% CI −0.13 to 1.91; Cohen *d*=0.22; *P*=.09), SAPS-Psychotic (mean difference −0.2, 95% CI −0.43 to 0.04, Cohen *d*=−0.20, *P*=.10), SANS (mean difference −0.14, 95% CI −0.33 to 0.04; Cohen *d*=−0.18; *P*=.13), SAPS-Disorganized (mean difference −0.02, 95% CI −0.16 to 0.11; Cohen *d*=−0.06; *P*=.71), GAF (mean difference 1.35, 95% CI −1.01 to 3.72; Cohen *d*=0.13; *P*=.26), PSP (mean difference 1.38, 95% CI −0.68 to 3.44; Cohen *d*=0.13; *P*=.19). There were no statistically significant differences between provider scores WAI-S Provider (mean difference −0.81, 95% CI −2.5, 0.87; Cohen *d*=−0.09; *P*=.34) or SES (MD=−0.10, 95% CI −0.48 to 0.28; Cohen *d*=−0.06; *P*=.60). Finally, we found no statistically significant difference between the intervention and control groups in the use of antipsychotic medication at the postintervention time point (odds ratio 0.46, 95% CI 0.13-1.61; *P*=.23).

Data from the Danish National Patient Register revealed no significant differences between the intervention and control groups in the mean number of hospitalizations (incidence rate ratio 0.80, 95% CI 0.27-2.37; *P*=.69) or length of admission in days (incidence rate ratio 0.76, 95% CI 0.11-5.53, *P*=.79). The incidence rate of hospitalization for the intervention group was 0.11 (95% CI 0.05-0.25), while that for the control group was 0.14 (95% CI 0.07-0.30). The incidence rate of days hospitalized for the intervention group was 1.60 (95% CI 0.39-6.56), while that of the control group was 2.10 (95% CI 0.52-8.46)

In terms of contacts to OPUS (eg, consultations), the intervention group had 2572 contacts (4.47 contacts per person per month) versus the control group having 2694 contacts (4.58 contacts per person per month), a nonsignificant difference.

**Table 3:** Intention-to-treat analyses of primary and secondary outcomes.

Intention-to-treat analyses	Intervention group					Control group					*P* value^a^			Cohen *d*
	Baseline, mean (SD)	Midintervention (3 months), mean (SD)	Postintervention (6 months), mean (SD)	Value, n (%)	Baseline, mean (SD)		Midintervention (3 months), mean (SD)	Postintervention (6 months), mean (SD)	Value, n (%)					
CHAI-MH^b^	55.52 (13.20)	61.04 (12.71)	64.91 (13.42)	96 (100)	56.49 (13.66)		61.33 (12.59)	61.19 (13.5)	98 (100)	.01		0.33		
PEPPI^c^	34.45 (8.40)	35.56 (8.85)	38.95 (7.13)	96 (100)	34.93 (8.7)		36.54 (7.36)	37.36 (8.1)	98 (100)	.05		0.24		
Hope	26.04 (9.04)	28.53 (6.99)	31.80 (7.36)	96 (100)	26.73 (9.12)		29.94 (7.71)	30.34 (9.29)	98 (100)	.12		0.20		
GSE^d^	23.02 (5.44)	24.47 (5.74)	26.74 (6.08)	96 (100)	23.79 (5.96)		25.70 (4.63)	26.10 (5.55)	98 (100)	.13		0.19		
WAI-S^e^	66.08 (10.78)	—^f^	69.21 (10.28)	96 (100)	66.56 (11.51)		—	67.20 (11.88)	98 (100)	.08		0.22		
PrepDM	58.33 (18.43)	—	66.84 (19.15)	96 (100)	62.57 (18.07)		—	64.58 (18.65)	98 (100)	.04		0.27		
CSQ^g^	26.27 (3.54)	—	27.34 (3.83)	96 (100)	26.63 (3.99)		—	26.76 (4.27)	98 (100)	.09		0.22		
Psychotic dimension^h^	2.06 (1.11)	—	1.42 (0.82)	96 (100)	2.02 (1.19)		—	1.59 (1.08)	98 (100)	.10		−0.20		
Negative dimension^i^	1.82 (0.92)	—	1.36 (0.81)	96 (100)	1.83 (0.93)		—	1.51 (0.77)	98 (100)	.13		−0.18		
Disorganized dimension^j^	0.53 (0.56)	—	0.31 (0.38)	96 (100)	0.62 (0.67)		—	0.38 (0.44)	98 (100)	.71		−0.06		
GAF^k^	56.14 (12.52)	—	62.39 (11.06)	96 (100)	53.84 (12.05)		—	59.34 (10.34)	98 (100)	.26		0.13		
PSP^l^	57.42 (12.23)	—	63.20 (10.78)	96 (100)	55.36 (11.76)		—	60.25 (10.10)	98 (100)	.19		0.13		
WAI-S (P)^m^	63.51 (9.30)	—	64.11 (9.30)	96 (100)	64.35 (8.44)		—	65.71 (8.25)	98 (100)	.34		−0.09		
SES^n^	2.36 (1.91)	—	2.34 (1.82)	96 (100)	2.00 (1.65)		—	1.96 (1.71)	98 (100)	.60		−0.06		

^a^Comparison of means between intervention group and control group postintervention.

^b^CHAI-MH: Consumer Health Activation Health Index–mental health version.

^c^PEPPI: Perceived Efficacy in Patient-Physician Interactions.

^d^GSE: General Self-Efficacy.

^e^WAI-S: Working Alliance Inventory–Short.

^f^Not available.

^g^CSQ: Client Satisfaction Questionnaire.

^h^Global item scores of hallucinations and delusions.

^i^Global item scores of affective flattering, alogia, avolition-apathy, and anhedonia.

^j^Global item scores of bizarre behaviors, formal thought disorder and single item score of inappropriate affect.

^k^GAF: Global Assessment of Functioning.

^l^PSP: Personal and Social Performance Scale.

^m^WAI-S (P): Working Alliance Inventory–Short (Provider version).

^n^SES: Service Engagement Scale.

**Table 4:** Intention-to-treat analyses of hospital admissions and use of medication.

Intention-to-treat analyses	Intervention group					Control group					*P* value^a^
	IRR^b^	IR^c^	Odds ratio (95% CI)	Value, n (%)	IRR		IR	Odds ratio (95% CI)	Value, n (%)		
Number of hospital admissions	0.80 (0.27-2.37)	0.11 (0.05-0.25)	—^d^	96 (100)	1 (reference)		0.14 (0.07-0.30)	—	98 (100)	.69	
Number of days admitted	0.76 (0.11-5.53)	1.60 (0.39-6.56)	—	96 (100)	1 (reference)		2.10 (0.52-8.46)	—	98 (100)	.79	
Use of medication	—	—	0.46 (0.13-1.61)	96 (100)	—		—	1 (reference)	98 (100)	.23	

^a^Comparison of means between intervention group and control group postintervention.

^b^IR: incidence rate.

^c^IRR: incidence rate ratio.

^d^Not available.

### Complete Case Analyses

Table 5 presents the results of the complete case analyses. The analyses showed similar results, although with larger variation, as in the ITT analyses; however, the statistically significant difference between groups on PEPPI was no longer present (mean difference 1.80, 95% CI −0.05 to 3.64, *P*=.06).

**Table 5:** Complete case analyses of primary and secondary outcomes.

Complete case analyses	Intervention group										Control group										*P* value^a^			Cohen *d*
	Baseline			Midintervention (3 months)			Postintervention (6 months)			Baseline				Midintervention (3 months)			Postintervention (6 months)							
	Value, mean (SD)	Value, n (%)	Value, mean (SD)		Value, n (%)	Value, mean (SD)		Value, n (%)	Value, mean (SD)			Value, n (%)	Value, mean (SD)		Value, n (%)	Value, mean (SD)		Value, n (%)						
CHAI-MH^b^	55.54 (13.27)	95 (98.96)	60.60 (13.45)		63 (65.63)	65.02 (13.88)		84 (87.5)	56.49 (13.66)			98 (100)	60.31 (13.67)		59 (60.20)	61.16 (13.77)		93 (94.90)	.01			0.31		
PEPPI^c^	34.49 (8.49)	94 (97.92)	35.82 (9.45)		61 (63.54)	39.07 (7.43)		82 (85.42)	35.01 (8.70)			97 (98.98)	36.76 (7.89)		58 (59.18)	37.17 (8.26)		92 (93.88)	.06			0.23		
Hope	26.04 (9.13)	94 (97.92)	28.50 (7.82)		60 (62.50)	31.87 (7.59)		84 (87.50)	26.74 (9.17)			97 (98.98)	28.95 (8.52)		57 (58.16)	30.33 (9.46)		91 (92.86)	.16			0.17		
GSE^d^	23.01 (5.51)	92 (95.83)	24.22 (6.13)		58 (60.42)	26.70 (6.56)		73 (76.04)	23.79 (5.96)			98 (100)	25.18 (5.25)		57 (58.16)	25.87 (5.67)		87 (88.78)	.12			0.19		
WAI-S^e^	66.08 (11.02)	91 (94.79)	—^f^		—	69.36 (10.53)		76 (79.17)	66.53 (11.67)			95 (96.94)	—		—	66.80 (12.42)		80 (81.63)	.09			0.20		
PrepDM	58.42 (18.81)	92 (95.83)	—		—	66.93 (20.37)		79 (82.29)	62.66 (18.15)			96 (97.96)	—		—	64.79 (19.47)		85 (86.73)	.04			0.26		
CSQ^g^	26.36 (3.60)	91 (94.79)	—		—	27.36 (3.99)		74 (77.08)	26.63 (3.99)			98 (100)	—		—	26.76 (4.52)		84 (85.71)	.09			0.20		
Psychotic dimension^h^	2.06 (1.11)	96 (100)	—		—	1.40 (0.94)		54 (56.25)	2.02 (1.19)			98 (100)	—		—	1.59 (1.12)		69 (70.41)	.15			−0.17		
Negative dimension^i^	1.82 (0.92)	96 (100)	—		—	1.17 (0.86)		53 (55.21)	1.83 (0.93)			98 (100)	—		—	1.51 (0.85)		65 (66.33)	.07			−0.20		
Disorganized dimension^j^	0.53 (0.56)	96 (100)	—		—	0.30 (0.43)		53 (55.21)	0.62 (0.67)			98 (100)	—		—	0.38 (0.46)		65 (66.33)	.49			−0.10		
GAF^k^	56.14 (12.52)	96 (100)	—		—	65.80 (11.35)		54 (56.25)	53.84 (12.05)			98 (100)	—		—	59.88 (11.17)		69 (70.41)	.20			0.13		
PSP^l^	57.42 (12.23)	96 (100)	—		—	66.56 (10.43)		54 (56.25)	55.36 (11.76)			98 (100)	—		—	60.78 (10.73)		69 (70.41)	.14			0.14		
WAI-S (P)^m^	63.55 (9.48)	92 (95.83)	—		—	64.09 (9.55)		90 (93.75)	64.34 (8.71)			92 (93.88)	—		—	65.88 (8.53)		86 (87.76)	.25			−0.11		
SES^n^	2.35 (1.94)	93 (96.88)	—		—	2.32 (1.86)		91 (94.79)	2.00 (1.70)			92 (93.88)	—		—	1.98 (1.80)		86 (87.76)	.55			0.06		
Use of medication^o^	—	96 (100)	—		—	—		54 (56.25)	—			98 (100)	—		—	—		69 (70.41)	.09			—		

^a^Comparison of means between intervention group and control group postintervention.

^b^CHAI-MH: Consumer Health Activation Health Index–mental health version.

^c^PEPPI: Perceived Efficacy in Patient-Physician Interactions.

^d^GSE: General Self-Efficacy.

^e^WAI-S: Working Alliance Inventory–Short.

^f^Not available.

^g^CSQ: Client Satisfaction Questionnaire.

^h^Global item scores of hallucinations and delusions.

^i^Global item scores of affective flattering, alogia, avolition-apathy, and anhedonia.

^j^Global item scores of bizarre behaviors, formal thought disorder and single item score of inappropriate affect.

^k^GAF: Global Assessment of Functioning.

^l^PSP: Personal and Social Performance Scale.

^m^WAI-S (P): Working Alliance Inventory–Short (Provider version).

^n^SES: Service Engagement Scale.

^o^There was no significant difference (*P*=.09) in the odds of using antipsychotic medication at the postintervention time point between the intervention group compared to the control group (OR 0.36, 95% CI 0.11-1.17).

### Explorative Outcomes

Although the aim of the study was to assess the effectiveness of the intervention after 6 months, we also assessed its effectiveness after 3 months for selected outcomes to explore when a potential effect would occur. On the basis of the ITT analyses and complete case analyses, we found no statistically significant differences between baseline and midintervention on CHAI-MH, PEPPI, Hope, or GSE.

Objective data on use of the app revealed that the intervention group had a mean use of 0.55 log-ins per day during their active use period (corresponding to roughly one session every second day). The active use period ranged from 1 day to 180 days, with a mean of 39 (SD 37.70) days, whereas the mean number of unique sessions was 23 ranging from 1 session to 148 sessions. When using the app, participants saw an average of 20 different screens, ranging from 5 to 28 screen views. Finally, 55% (47/96) of participants in the intervention group logged in after the first month. On the basis of the App Rating Questionnaire, participants were somewhat satisfied with the app (mean score was 6.36 out of 12), while they rated the app to be of average quality (mean score was 2.85 out of 5).

## Discussion

### Principal Findings

This study presents the results of a clinical trial investigating a digital SDM tool to promote patient activation for people diagnosed with schizophrenia. The study found a statistically significant difference in our primary outcome, patient activation, CHAI-MH (mean difference 4.39, 95% CI 0.99-7.79; Cohen *d*=0.33; *P*=.01), favoring the intervention group. These findings confirm our hypothesis that a digital SDM tool may promote patient activation by supporting the collaborative process between patients and their providers and is in concordance with recent meta-analyses on the effectiveness of digital SDM interventions that found these types of interventions to have an effect on patient activation [11]. The effect size (Cohen *d*) for patient activation was 0.33, which may be interpreted as a small effect size. According to our protocol and power calculations, we expected to find an effect size of 0.42, thereby not reaching the anticipated effect. In addition, it is unclear whether this effect size is clinically relevant. In somatic care, patient activation has been found to play an important role in improving quality and health outcomes, where every 10 points in patient activation were associated with a 1% decreased probability of visiting an emergency unit, being obese, or smoking [29]. The mean difference in our trial was 4.39 and somewhat far off the 10 points found in the study by Greene and Hibbard [29]. However, such studies have not been conducted in mental health care and are needed to better assess the minimal clinical relevance of people with a mental health condition having higher levels of patient activation. However, as argued in the trial by Hamann et al [2], SDM interventions can improve the feeling of being involved in one’s treatment, which may be particularly useful for people feeling involuntarily treated or those who refuse treatment due to a lack of insight in their care.

Our intervention also had an effect on 2 secondary outcomes: PEPPI and PrepDM. Although these results were close to the 0.05 cut-off level, they favored the intervention group, similar to our primary outcome. In addition, for the complete case analyses, we found a statistically significant difference in PrepDM, favoring the intervention group. Thus, our trial indicates that a digital SDM tool is effective in improving patient activation, feeling prepared for decision-making, and confidence in communicating with one’s provider.

Although none of the other secondary outcomes had a statistically significant effect, most secondary outcomes favored the intervention group. One unexpected finding was that we did not find a statistically significant effect on satisfaction since SDM has been strongly advocated as a process to increase patient satisfaction with treatment. However, similar to other trials, we encountered a ceiling effect on the CSQ scale, with 44.8% (87/194) of participants scoring ≥29 out of 32 [30].

Despite the difference in self-perceived patient activation, we found no difference in how providers perceived their patients’ level of engagement via the SES (mean difference −0.10, 95% CI −0.48 to 0.28; Cohen *d*=−0.06; *P*=.60). It may be intuitive to assume that increased levels of patient activation are associated with an increase in providers’ perceptions of patients’ level of engagement. However, studies have found that some providers find it challenging when patients become more active and ask questions that the provider might not always have an answer to [31]. This highlights that while promoting patient activation may be beneficial for the patient, it may also be important to consider how the provider responds to a suddenly more active and engaged patient and whether the provider needs support in adapting to this change. Another potential explanation for why providers seemingly did not report a difference in the group level of activation could be that the mean difference between the groups’ CHAI-MH scores was too small for the providers to distinguish.

Although our intervention was a digital SDM tool, we did not include a specific SDM outcome measurement. This is due to our conceptual definition of SDM, defining SDM as a process rather than an outcome, and the limitation of relevant SDM measurements. While specific SDM measurements have been developed, many of these measurements are focused on a concrete decision (eg, my provider and I chose a treatment option together) rather than on the process of SDM. Challenges in measuring SDM have previously been identified, and measurements to evaluate the SDM process with adequate psychometric properties are needed [32].

On the basis of the explorative midintervention assessment, the difference in patient activation between the groups occurred between the mid- and postintervention assessment. Each group had a similar increase in CHAI-MH score from baseline to the midintervention assessment, with no between-group differences. However, only the intervention group continued to increase their CHAI-MH scores from during the intervention to after the intervention, resulting in a statistically significant difference between the 2 groups. This may indicate that the effect of a digital SDM tool may not occur quickly but instead requires time to develop an effect. What seems contradictory is that data on app use indicate that approximately half of the participants stopped using the app after 1 month. These explorative findings suggest that participants who stopped using the app before the end of the intervention may still have benefited from it.

Our intervention group encountered both barriers and difficulties in acquiring and using an app in combination with their treatment. First, 10 participants were invited to use the app but never open it. Although we did not explore the reasons for this, there were reports of technical limitations (eg, the phone system did not support the app). The study was also affected by the fire accident at OVHcloud. Around half of the users who had used the app before the accident did not log in after the system became available again, while the mean use of the app also decreased. The magnitude of such accident is rare but does highlight a vulnerability to digital systems while also highlighting a challenge in re-engaging participants after a “pause” from an intervention. It also questions whether the effect of the digital SDM tool could have been greater if these limitations had been avoided.

### Strengths and Limitations

This study had several strengths. First, we included and assessed both patients and their providers to acknowledge the importance of both in the process of SDM. Second, the study had a large sample size with a low level of lost-to-follow-up on our main outcome. Third, the study had a pragmatic nature, in which the use of the system would be similar to how it would be used in practice outside of the trial. Therefore, the results should be generalizable to other similar services.

However, this pragmatic approach is limited in terms of support for participants. Participants who encountered an issue with the app were instructed to ask their provider for assistance who were then able to consult with a blinded student assistant or an IT supporter. This placed a large responsibility on the provider. If the provider did not resolve the situation or contact support, the patient could be prone to stop using the app. A recent study highlighted that with the rapid development and use of digital tools in mental health care, educational efforts are needed to strengthen the clinician’s knowledge and skills regarding these tools [33]. Future trials investigating a digital system are encouraged to carefully consider how participants (patients and providers) are supported in the case of issues or barriers.

During our recruitment, we randomized patients to either the control or intervention group, meaning that providers were able to have patients in both groups. This creates a risk for a contamination effect, as providers were able to use elements from the intervention with patients in the control group. One way to address this would have been to randomize at the clinician or clinic level to avoid providers having participants in each group. Doing so would potentially have made it more difficult to recruit participants unless they could have been to assign patients to a waitlist. However, this was not possible in this trial.

This recruitment process may challenge the generalizability of the study. The vast majority of participants were recruited by providers to inform patients about the study. Although providers were strongly encouraged to ask all of their patients about the research project, providers were able to, on their own, select which patients to inform about the study. Providers may have been more prone to ask patients they assume would use a smartphone app or patients whom the provider believed were able to participate in such a trial. This recruitment process may have affected the distribution of the study participants’ characteristics, such as gender, diagnosis, or use of antipsychotic medication. For example, the level of functioning of the included participants was significantly higher in our sample than in a sample from a previous OPUS project [34]. Furthermore, this selection by the provider may show a lack of SDM between the patient and provider in which the provider decides whether to inform the patient about the research project, thereby not giving the patient a say when making the decision about participating in the study.

During the trial, researchers routinely made providers aware of the project by being physically present at the clinic. However, the COVID-19 pandemic added another challenge, in which it was not possible to be as present at the clinic as usual. However, with the pandemic, health care and many other areas saw an increased use of digital systems and how quickly we can adopt these systems into practice. eHealth provides an approach to care when in-person services are troublesome [33]. Moving forward, stakeholders and practitioners are encouraged to adopt e–mental health care tools to offer a more blended care plan [35].

Although the level of lost-to-follow-up on our primary outcome was low (<10%), we observed that the intervention group had a higher number at the postintervention time point than the control group, which may have biased our results. In addition, the higher number of participants lost to follow-up in the intervention group may be caused by boredom or dissatisfaction with the tool. However, disengagement can also be interpreted as a potentially harmful outcome of using a digital SDM tool. However, the absolute numbers of lost-to-follow-up were relatively low, and the reasons in the intervention group were mostly due to ending OPUS treatment prematurely. Another potential bias in the trial was the fact that a majority of outcomes (including the primary outcome) were self-reported, and as participants were not blinded, this could introduce a bias by overestimating the true effect size.

### Conclusions

The Momentum Trial had a significant beneficial effect on the primary outcome, patient activation, at the postintervention time point (mean difference was 4.39 point favoring the intervention group with 95% CI 0.99-7.79; Cohen *d*=0.33; *P*=.01). The effect size was smaller than the 0.42 effect size that we had anticipated and sampled for. The intervention was also effective in improving secondary outcomes: confidence in communicating with one’s provider and feeling prepared when making treatment decisions. Despite our hypothesis, the Momentum Trial had no effect on hope, treatment satisfaction, working alliance, or clinical outcomes.

The Momentum Trial strengthens the existing evidence by demonstrating that digital SDM interventions can be effective in supporting patients to feel active and engaged in their treatment. This intervention had important limitations that should be considered in future trials.

## Appendix

Multimedia Appendix 1 CONSORT-EHEALTH Checklist (V 1.6.1).

## Abbreviations

CDMS: Clinical Decision Making Style
CHAI-MH: Consumer Health Activation Index for mental health
CONSORT: Consolidated Standards of Reporting Trials
CSQ: Client Satisfaction Questionnaire
GAF: Global Assessment of Functioning
GSE: General Self-Efficacy Scale
ITT: intention to treat
OPEN: Odense Patient Data Explorative Network
PEPPI: Perceived Efficacy in Patient-Physician Interactions
PrepDM: Preparation for Decision-Making
PSP: Personal and Social Performance Scale
SANS: Scale for the Assessment of Negative Symptoms
SAPS: Scale for the Assessment of Positive Symptoms
SES: Service Engagement Scale
SDM: shared decision-making
TAU: treatment as usual
WAI-S: Working Alliance Inventory–short form

## Data Availability

The data generated from the trial are available from the corresponding author upon reasonable request.
